# Regorafenib enhances antitumor immunity via inhibition of p38 kinase/Creb1/Klf4 axis in tumor-associated macrophages

**DOI:** 10.1136/jitc-2020-001657

**Published:** 2021-03-22

**Authors:** Da-Liang Ou, Chia-Wei Chen, Chia-Lang Hsu, Chih-Hung Chung, Zi-Rui Feng, Bin-Shyun Lee, Ann-Lii Cheng, Muh-Hwa Yang, Chiun Hsu

**Affiliations:** 1 Graduate Institute of Oncology, National Taiwan University College of Medicine, Taipei, Taiwan; 2 Department of Medical Research, National Taiwan University Hospital, Taipei, Taiwan; 3 Graduate Institute of Medical Genomics and Proteomics, National Taiwan University College of Medicine, Taipei, Taiwan; 4 Taiwan International Graduate Program in Molecular Medicine, National Yang-Ming University, Taipei, Taiwan; 5 Department of Oncology, National Taiwan University Hospital, Taipei, Taiwan; 6 National Taiwan University Cancer Center, Taipei, Taiwan; 7 Department of Internal Medicine, National Taiwan University Hospital, Taipei, Taiwan; 8 Institute of Clinical Medicine, National Yang-Ming University, Taipei, Taiwan; 9 Division of Medical Oncology, Department of Oncology, Taipei Veterans General Hospital, Taipei, Taiwan

**Keywords:** immunomodulation, immunotherapy, tumor microenvironment

## Abstract

**Background:**

Regorafenib and other multikinase inhibitors may enhance antitumor efficacy of anti-program cell death-1 (anti-PD1) therapy in hepatocellular carcinoma (HCC). Its immunomodulatory effects, besides anti-angiogenesis, were not clearly defined.

**Methods:**

In vivo antitumor efficacy was tested in multiple syngeneic liver cancer models. Murine bone marrow–derived macrophages (BMDMs) were tested in vitro for modulation of polarization by regorafenib and activation of cocultured T cells. Markers of M1/M2 polarization were measured by quantitative reverse transcription PCR (RT-PCR), arginase activity, flow cytometry, and ELISA. Knockdown of p38 kinase and downstream Creb1/Klf4 signaling on macrophage polarization were confirmed by using knockdown of the upstream MAPK14 kinase, chemical p38 kinase inhibitor, and chromatin immunoprecipitation.

**Results:**

Regorafenib (5 mg/kg/day, corresponding to about half of human clinical dosage) inhibited tumor growth and angiogenesis in vivo similarly to DC-101 (anti-VEGFR2 antibody) but produced higher T cell activation and M1 macrophage polarization, increased the ratio of M1/M2 polarized BMDMs and proliferation/activation of cocultured T cells in vitro, indicating angiogenesis-independent immunomodulatory effects. Suppression of p38 kinase phosphorylation and downstream Creb1/Klf4 activity in BMDMs by regorafenib reversed M2 polarization. Regorafenib enhanced antitumor efficacy of adoptively transferred antigen-specific T cells. Synergistic antitumor efficacy between regorafenib and anti-PD1 was associated with multiple immune-related pathways in the tumor microenvironment.

**Conclusion:**

Regorafenib may enhance antitumor immunity through modulation of macrophage polarization, independent of its anti-angiogenic effects. Optimization of regorafenib dosage for rational design of combination therapy regimen may improve the therapeutic index in the clinic.

## Introduction

The program cell death-1 (PD-1)/program death ligand-1 (PD-L1) pathway has been extensively studied for its role in regulation of antitumor immunity.^1^ Single-agent anti-PD-1 or anti-PD-L1 immune checkpoint inhibitor (ICI) therapy has been approved for more than 10 types of advanced cancers, and combination regimens with other immunomodulatory agents, targeted therapy, or cytotoxic chemotherapy may further improve overall survival (OS) or progression-free survival (PFS) in different cancer types. For patients with advanced hepatocellular carcinoma (HCC), the combination of atezolizumab (anti-PD-L1 ICI) and bevacizumab (anti-angiogenic agent) demonstrated superior OS, PFS, and objective tumor response, compared with the multikinase inhibitor sorafenib, and establishes a new standard of first-line systemic therapy for advanced HCC.^2^

The immune modulatory effects of anti-angiogenic therapy has been extensively studied by both preclinical models and clinical trials.^3–5^ For HCC, anti-angiogenic effect via inhibition of vascular endothelial growth factor (VEGF) pathway is a common antitumor mechanism of all the approved MKIs (sorafenib and lenvatinib in the first line; regorafenib and cabozantinib in the second line), and combination of these MKIs with ICI for HCC therapy is extensively studied.^6^ A caveat of developing this type of combination regimens is the safety issue, as dose reduction of MKI and treatment discontinuation due to adverse events are common and have led to discontinuation of development of some ICI plus anti-angiogenic combinations.^7–10^ Moreover, preclinical studies suggested that higher dosage of MKI may paradoxically induce immunosuppression through induction of hypoxia and recruitment of tumor-associated macrophages (TAMs) or other suppressive cells.^11^ Identification of the optimal immune modulatory effects of targeted agents is thus critical for development of combination immunotherapy both to improve the therapeutic index and to tailor the use of targeted agents to their biologically effective and clinically relevant dosage.

The MKI regorafenib has been approved as second-line therapy for advanced HCC, as well as refractory colorectal cancer and gastrointestinal stromal tumor, but its use at the recommended dosage (160 mg per day for 21 days followed by 7-day rest) is frequently limited by prominent treatment-related adverse events, including hand-foot skin reaction, diarrhea, and hypertension. Combination of regorafenib and the anti-PD-1 agent nivolumab demonstrated an objective response rate of 40% in 50 patients with heavily pretreated advanced gastric or colorectal cancers, for whom the response rates of either regorafenib or nivolumab alone were lower than 10%.^12^ Regorafenib at 80 mg/day was well tolerated in this combination, while the recommended dosage (160 mg/day) produced prominent skin toxicity and other adverse events without increase in efficacy. We and other investigators have demonstrated that regorafenib at sub-micromolar range may induce M1 macrophage polarization and increased proliferation and activation of CD8+ T cells. In vivo studies using low-dose regorafenib 3–5 mg/kg/day, corresponding to about 50% of the single-agent recommended dosage, demonstrated synergistic antitumor efficacy with anti-PD-1 therapy.^13 14^ The above observations indicate that the optimal immune modulatory dosage of regorafenib may be lower than recommended for single-agent therapy and should be optimized to improve its safety profile and facilitate development in combination therapy.

This study sought to establish relevant preclinical models to characterize the immune modulatory effects of regorafenib and to explore the biologically effective dosage of regorafenib for HCC. Low-dose regorafenib (≤1 µM in vitro, 5 mg/kg/day in vivo) may increase cytotoxic T cell function and antitumor immunity through polarization of macrophages toward the M1 phenotype. Furthermore, the p38 mitogen-activated protein kinase (p38MAPK) pathway was identified as one molecular mechanism mediating the immune modulatory effects of regorafenib.

## Materials and methods

### Murine liver cancer models, cell lines, and reagents

The protocol for the animal experiments was approved by the Institutional Animal Care and Use Committee (IACUC) of the College of Medicine, National Taiwan University, and conformed to the criteria outlined in the Guide for the Care and Use of Laboratory Animals (BALB/c and C57BL/6 mice from National Laboratory Animal Center, Taiwan; P14 transgenic mice (C57BL/6J background) from Jackson Laboratories were housed and kept under specific pathogen-free conditions at the laboratory animal center in National Taiwan University College of Medicine).

The details of human and murine liver cancer and macrophage cell lines were listed in the supplemental file. In vitro cell viability was measured using an MTT (3-(4,5-dimethylthiazol-2-yl)-2,5-diphenyltetrazolium bromide) assay or sub-G1 fraction analysis (for apoptotic cells) using flow cytometry as previously described.^15^ For the subcutaneous model (Hepa1-6 cell line/C57BL/6 mice), about 2×10^6^ cells were injected subcutaneously into the right flank, and drug treatment was started when tumor volume was about 100 mm^3^ (calculated by the formula 0.5×length×width^2^) and the mice were randomized into each treatment group. For the orthotopic model (BNL cell line/BALB/c mice), about 2×10^5^ cells were injected into the subcapsular area of the left liver lobe, and the mice were randomized to each treatment group 5 days after tumor cell injection, based on our previous study.^16^ The schema of animal experiments was provided in the supplemental file.

The antitumor efficacy and safety of drug treatment were measured by change in tumor volume, body weight, and animal survival.

To measure antigen-specific antitumor immunity, Hepa1-6 liver cancer cells were transfected with a vector that overexpressed a glycoprotein epitope (GP33) of the lymphocytic choriomeningitis virus (a generous gift from Professor Dr Hanspeter Pircher, University of Freiburg).^17^ Lymphocytes from P14 transgenic mice, which specifically recognized GP33 expressed on Hepa1-6 cells, were used in experiments of adoptive transfer.

Regorafenib was provided by Bayer (Bayer AG, Berlin, Germany). Details of other reagents were listed in the supplemental file. In animal studies, regorafenib was dissolved in polypropylene glycol, PEG400, Pluronic F68, water (34:34:12:20) and given orally by gavage.^18^ The anti-VEGFR antibody DC-101 was given by intraperitoneal injection.

### In vitro modulation of macrophage polarization

Mouse BMDMs were prepared as described in the supplemental file. BMDMs or J774A.1 cells were polarized with interferon-γ (IFN-γ, 20 ng/mL, R&D)+lipopolysaccharide (LPS, 50 ng/mL, Sigma) or interleukin-4 (IL4) (20 ng/mL, R&D) for 24 hours to induce M1 or M2 phenotypes, respectively. Markers of M1 (TNF-α, IL-6, MHC II) or M2 (Arginase-1, CD206) phenotypes were measured by quantitative reverse transcription PCR (RT-PCR), arginase activity, flow cytometry, and ELISA (Invitrogen) analysis. Murine splenocytes were cocultured with BMDMs, and CD8+/CD4+ T cell function modulated by macrophages with or without drug treatment was measured by T cell proliferation (carboxyfluorescein succinimidyl ester (CFSE) staining, Invitrogen) and IFN-γ secretion (Mouse IFN-γ ELISA kit, Invitrogen).

The key signaling pathways in BMDMs modulated by regorafenib were screened by the phospho-kinase array (R&D systems) according to the manufacturer’s instructions and confirmed by western blotting. Knockdown of p38 kinase was performed using lentivirus transduction of green fluorescence protein (GFP) control and MAPK14 clones (from the National RNAi Core Facility, Academia Sinica, Taiwan) into J774A.1 cells. The GFP+ cells were sorted by fluorescence-activated cell sorting (FACS) to increase the proportion of successfully transduced cells and the changes in relevant signaling molecules in sorted cells were measured by western blotting. The modulatory effects on signaling and associated downstream transcriptional regulation of the immune regulatory factor Krupple-like factor 4 (Klf4) by cAMP responsive element binding protein 1 (Creb1) by regorafenib was further measured by chromatin immunoprecipitation (ChIP, see the supplemental file for detailed primer design and experiment procedures).

### RNA-sequencing and bioinformatics analysis

Expression of immune-related genes was measured by RNA sequencing using the Illumina Nextseq 500 system (Illumina), with a read length of 2×100 bases (Genomics, Taipei, Taiwan). Initial quality control was performed using FastQC (V.0.11.8) and removal of adaptors was performed using cutadapt (V.2.4). The qualified reads were mapped to the mouse genome (GRCm38) using STAR (V.2.7.2a). Gene-level read counts were obtained using STAR with parameter ‘—quantMode GeneCounts’ based on the gene definition of Gencode (V.M19).

Read counts were normalized by Trimmed Mean of M-values (TMM) method implemented by edgeR (V.3.28.0). If the datasets had biological replicates, differential expression analysis was performed by R package limma (V.3.42.2), otherwise by NOISeq (V.2.30.0) with no replicate mode. Gene-set enrichment analysis (GSEA) and over-representation analyses were performed using the functions implemented by clusterProfiler (V.3.14.3). The gene sets were collected from MSigDB (V.7.0) including the category C2 and C5, as well as manually curated from the literature (online supplemental table S1). The ranking metrics of signal to noise and log2 ratio were used for dataset with/without biological replicates, respectively. The enrichment map was constructed as previously described.^19^ The gene sets were linked if the arithmetic mean of Jaccard and overlap coefficients of two gene sets is larger than 0.3.

10.1136/jitc-2020-001657.supp1 Supplementary data

### Regulation of tumor microenvironment by regorafenib in vivo

The immune microenvironment of murine tumors (orthotopic or heterotopic) after drug treatment was evaluated by changes in immune regulatory genes (RNA-seq and GSEA) and immune cell composition (flow cytometry, immunohistochemistry, and multiplex immunofluorescence staining). Fresh tumor samples were used for RNA-seq (using the procedures described above) and flow cytometry analysis. For flow cytometry analysis of tumor-infiltrating immune cells, tumor tissue was dissociated using gentleMACS Dissociator (Miltenyi Biotec) and isolated by density gradient centrifugation. Cell suspensions were stained with antibodies against surface markers or, for intracellular staining, stained with antibodies against intracellular markers after permeation and fixation. The results were analyzed by LSRFortessa (BD Bioscience) flow cytometry and the FlowJo V.10 software.

Formalin-fixed, paraffin-embedded (FFPE) tumor sections were used to measure the levels of tumor-infiltrating T cells, angiogenesis, cell proliferation (by immunohistochemical staining), and apoptosis (by terminal deoxynucleotidyl transferase dUTP nick end labeling (TUNEL) assay (Promega)). The Polaris system (PerkinElmer, Waltham, Massachusetts, USA) and the Opal 4-Color Manual IHC Kit (PerkinElmer, NEL810001KT) were used to detect F4/80, MHC II, and CD206 expression in FFPE sections (see the supplemental file for detailed procedures and the antibody list). Multispectral images for each tumor tissue section were acquired using the Phenochart and the inForm software (PerkinElmer). To train the phenotype classifier and create a tissue segmentation algorithm by the inForm software, 10 representative tumor images were selected as the training set and manually annotated for M1 (F4/80+MHC II+CD206-) or M2 (F4/80+MHCII-CD206+) macrophages. Cell segmentation was performed based on the nuclear DAPI (4′,6-diamidino-2-phenylindole) staining. Tissue and cell segmentation algorithms were then established for cell phenotyping and measurement of immune cell composition through machine-learning by the inForm software.

The potential effects of regorafenib on antigen-specific antitumor immunity were evaluated using adoptive transfer of lymphocytes from P14 transgenic mice to treat tumors established by GP33-expressing Hepa1-6 cells. CD8+ T cells isolated from P14 transgenic mice, using the EasySep Mouse CD8a Positive Selection Kit II (STEMCELL), were activated using anti-CD3 (2 µg/mL), anti-CD28 (2 µg/mL), and LCMV gp33-41 (1 µM) for 72 hours following CFSE labeling. About 1×10^7^ CFSE-labeled P14 CD8+ T cells or phosphate-buffered saline (PBS) were injected, via orbital vascular plexus, into control or regorafenib-treated C57BL/6 mice bearing Hepa1-6-gp33 cells. Regorafenib (5 mg/kg/day) was administrated for 5 days before adoptive transfer. Treatment with anti-mouse PD1 antibody (clone RMP1–14), or isotype control antibody (clone 2A3) (Bio X Cell, West Lebanon, New Hampshire, USA), 0.2 mg per dose, was given on days 5, 6, 9, 12, and 15 after tumor implantation by intraperitoneal injection.

### Statistical analysis

All data were representative of at least three independent experiments. Quantitative data were represented as mean±SD or SE of the mean (SEM), as indicated in the legend. Two-tailed Student’s t-test with equal variance was performed to compare two experimental groups. One-way analysis of variance (ANOVA) with Tukey’s post hoc test was performed to evaluate the difference between multiple groups. Repeated-measures ANOVA was performed to evaluate the tumor growth curves, and survival analysis was measured using the Kaplan-Meier method and analyzed by log-rank test. All the statistical analyses were performed using SPSS V.21 or Office Excel V.2019. The raw data of RNA-seq were all available in GEO (accession number GSE148950).

## Results

### The immune modulatory effects of regorafenib contribute to its in vivo antitumor efficacy

**Figure 1:**
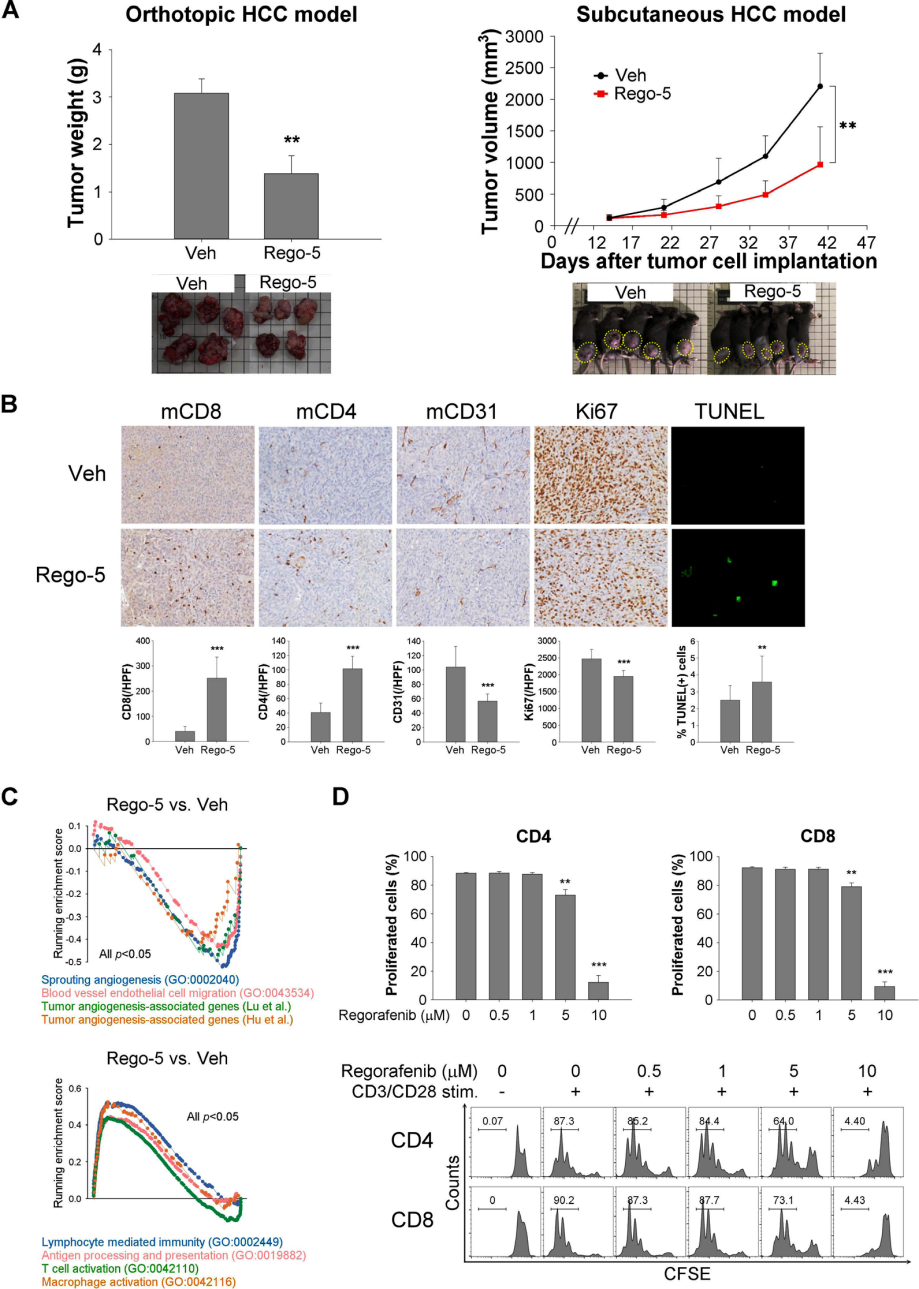
The immune modulatory effects of regorafenib contribute to its in vivo antitumor efficacy. (A) The in vivo antitumor efficacy of regorafenib in immune-competent (BNL-MEA cells implanted orthotopically into BALB/c mice (N=5) and Hepa1-6 cells implanted subcutaneously into C57BL/6 mice (N=10)) liver cancer models. Mice were treated with vehicle, regorafenib 5 mg/kg/day (Rego-5) for 28 days and the tumor weight and volume were monitored. (B) Tumor-infiltrating T cells (CD4 and CD8), tumor cell proliferation (Ki67), and tumor angiogenesis (CD31) were quantified by immunohistochemical staining. Apoptotic tumor cells were quantified by terminal deoxynucleotidyltransferase dUTP nick end labeling (TUNEL) assay. Tumor samples were collected from BNL-MEA tumor-bearing BALB/c mice treated after 5 days of treatment with vehicle or regorafenib 5 mg/kg/day by gavage. Data were analyzed using 20 images (regions of interest, ROI)/ tumor, 4 tumors from 4 mice in each treatment group. (C) Gene-set enrichment analysis (GSEA) of leukocyte activation and angiogenesis signatures in RNA of tumor bulk from BNL-MEA tumor-bearing BALB/c mice treated with vehicle or regorafenib for 5 days. (D) Murine splenocytes from BALB/c mice were treated regorafenib (0, 0.5, 1, 5, 10 μM) in vitro and the proliferation of CD4+ and CD8+ T cells was detected with carboxyfluorescein succinimidyl ester (CFSE) staining and flow cytometry. Data are presented as the mean±SD from a representative experiment of at least triplicate. *p<0.05; **p<0.01; ***p<0.001, two-tailed Student’s t-test. HCC, hepatocellular carcinoma.

Regorafenib 5 mg/kg/day, corresponding to about 50% of the recommended dosage for human based on clinical and preclinical pharmacokinetic data,^20^ exerted antitumor efficacy in both orthotopic and subcutaneous immune-competent liver cancer models (figure 1A). The difference in tumor growth measured at the end of the animal study may reflect the combined effects of decreased cell proliferation (Ki67 staining), decreased tumor angiogenesis (CD31 staining), and increased tumor cell apoptosis (TUNEL assay) by regorafenib (figure 1B). Regorafenib increased CD4+ and CD8+ T cell infiltration into tumors, increased expression of genes related to T cell activation, antigen presentation, and macrophage activation, and suppressed genes related to angiogenesis (figure 1B, C,online supplemental table S2). While regorafenib induce cancer cell apoptosis dose dependently (online supplemental figure S1A, B), higher dosage of regorafenib inhibited T cell proliferation and activation in vitro (figure 1D, online supplemental figure S1C), suggesting that higher dosage of regorafenib may not be associated with better immune modulatory effects.

10.1136/jitc-2020-001657.supp2 Supplementary data

**Figure 2:**
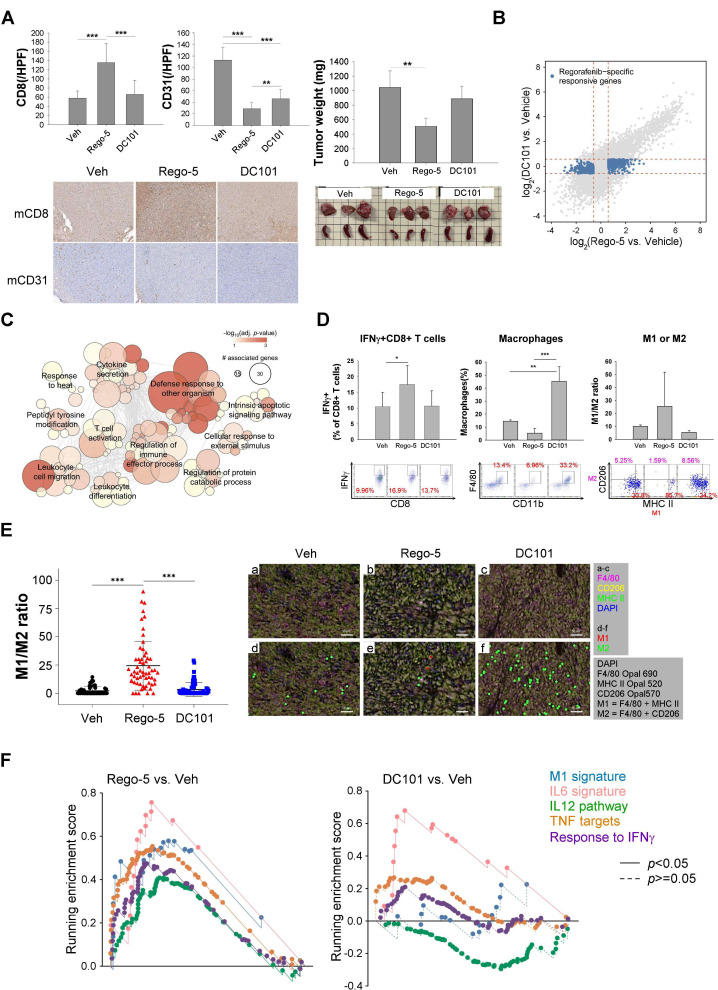
Regorafenib had immunomodulatory effects, which are independent of anti-angiogenesis. (A) Comparison between regorafenib and DC-101, a murine anti-VEGFR2 antibody, in terms of induction of CD8+ T cell infiltration (CD8 staining), angiogenesis inhibition (CD31 staining), and antitumor efficacy. BALB/c mice implanted BNL-MEA cells orthotopically were treated with regorafenib (5 mg/kg/day) or DC-101 (800 μg, intraperitoneal, days 1, 3, 5) for 5 days. (B) Scatter plot comparing the fold changes of genes regulated by regorafenib or DC-101. The blue dots indicated genes whose regulations were considered independent of VEGFR2 inhibition. (C) Enrichment map showing GO terms of regorafenib-regulated, VEGFR2-independent genes. Groups of functionally related gene sets were highlighted. (D) Composition of tumor-infiltrating immune cells analyzed by flow cytometry. Orthotopic liver tumor samples were collected 5 days after treatment start and the percentage of individual immune cell types was measured by flow cytometry. Values are presented as means±SD (n=3 in each group). (E) Regulation of macrophage polarization by regorafenib in vivo, indicated by the change in the ratio of M1 (F4/80+MHCII+CD206-))/M2 (F4/80+MHCII-CD206+) cells measured by multiplex immunofluorescence staining. Multispectral images were acquired to cover the whole area of the specimens; each dot in the left panel represented one acquisition region of interest (ROI). Data were analyzed using 60 ROI images/ tumor, 4 tumors from 4 mice in each treatment group. Right panel, representative spectrally unmixed composite images (×20 magnification) from the multiplex immunofluorescence staining. (F) Gene-set enrichment analysis (GSEA) of signaling pathways related to macrophage activation in tumors treatment with regorafenib or DC-101 compared with vehicle. *, p < 0.05; **, p < 0.01; ***, p < 0.001.

The antitumor effects of regorafenib were then compared with those of DC-101, a murine anti-VEGFR2 antibody, in an orthotopic liver cancer model to identify possible immune modulatory effects independent of anti-VEGFR2 effects. While both regorafenib and DC-101 inhibited tumor angiogenesis, regorafenib induced more T cell infiltration into the tumors and was associated with better antitumor effects (figure 2A, supplementary figure S2A). Regorafenib regulated multiple immune-related pathways that were relatively unaffected by VEGFR2 inhibition, such as those related to defense response and leukocyte migration (figure 2B, C, online supplemental table S3, S4), and increased activated CD8+ T cells in the tumors (figure 2D, supplementary figure S2B-D). Regorafenib decreased the total number of TAMs in vivo (figure 2D) and induced apoptosis of M1/M2 macrophages similarly in vitro (online supplemental figure S3). However, both flow cytometry and multiplex immunofluorescence staining studies disclosed increased M1/M2 ratio (figure 2D, E). By contrast, DC-101 increased the total numbers of TAMs and did not affect macrophage polarization.

We thus hypothesized that regulation of macrophage polarization may account for the immune modulatory effects of regorafenib, independent of its VEGFR2 inhibitory effects. This hypothesis was supported by the differential effects of regorafenib and DC-101 on regulation of key signaling pathways for macrophage function. Regorafenib induced more prominent changes in the M1 signature,^21^ IL-12 pathways, response to IFN-γ, and targets of tumor necrosis factors (TNFs) (figure 2F, online supplemental table S5).^22^ Effects of regorafenib and DC-101 did not differ significantly in other immune cells analyzed, such as dendritic cells, regulatory T cells, or myeloid-derived suppressor cells (online supplemental figure S2C).

### Regorafenib increased proliferation and activation of CD8+ T cells via regulation of macrophage polarization

**Figure 3:**
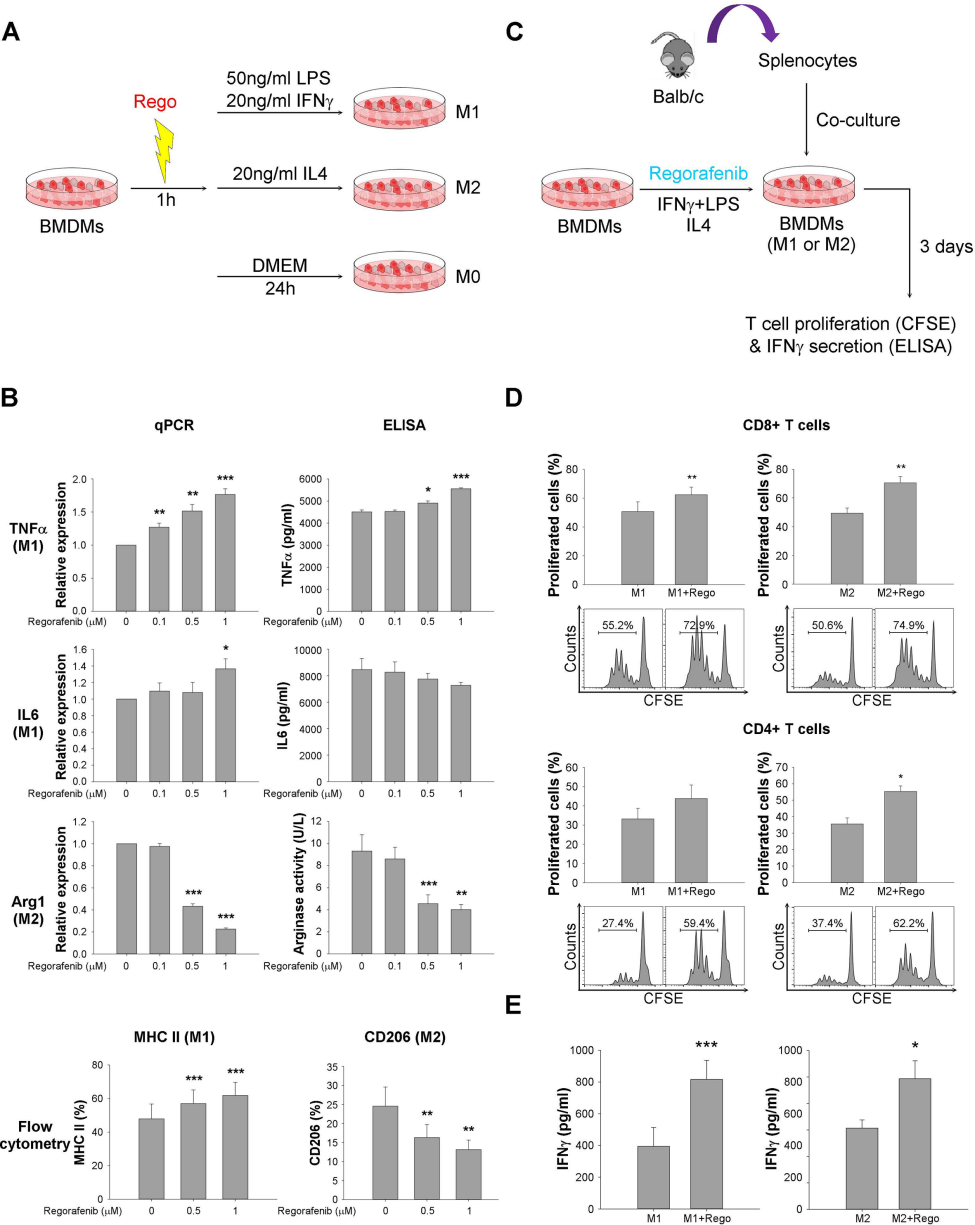
Regorafenib increased proliferation and activation of CD8+ T cells via regulation of macrophage polarization. (A) Design of in vitro study to measure the impact of regorafenib on macrophage polarization. Bone marrow–derived macrophages (BMDMs) were pretreated with regorafenib for 1 hour and then polarized to by interferon-γ (IFNγ)+lipopolysaccharides (LPS) (M1 phenotype) or interleukin-4 (IL4) (M2 phenotype), respectively for 24 hours. M1 (TNFα, IL6, MHC II) and M2 (Arg1, CD206) markers were detected by qPCR, ELISA, flow cytometry, and arginase activity. (B) Expression of M1 markers was enhanced by regorafenib, while expression of M2 markers was suppressed. (C) Design of coculture study to measure the impact of regorafenib-treated BMDMs on T cell function. BMDMs treated with/without regorafenib (1 µM) were cocultured with carboxyfluorescein succinimidyl ester (CFSE)-labeled murine splenocytes at 20:1 ratio for 72 hours. (D) T cell proliferation, measured by CFSE staining and flow cytometry, was enhanced by regorafenib-treated BMDMs. (E) T cell activation, measured by IFN-γ secretion into culture medium using ELISA, was enhanced by regorafenib-treated BMDMs. *, p < 0.05; **, p < 0.01; ***, p <0.001.

To measure the effects of regorafenib on macrophage polarization and T cell function in vitro, BMDMs were pretreated with regorafenib for 1 hour and then polarized by IFN-γ+lipopolysaccharides (LPS) (M1 phenotype) or by interleukin-4 (IL4) (M2 phenotype), respectively, for 24 hours (figure 3A). Regorafenib at sub-micromolar dosages increased M1 markers (TNFα, IL6, MHC II) in M1 macrophages and suppressed M2 markers (Arg1, CD206) in M2 macrophages (figure 3B). Coculture of regorafenib-treated BMDMs (figure 3C) increased T cell proliferation and IFN-γ secretion (figure 3D, E). The above data suggested that regulation of macrophage function, especially suppression of M2 polarization, may play key roles in the immune modulatory effects of regorafenib.

**Figure 4:**
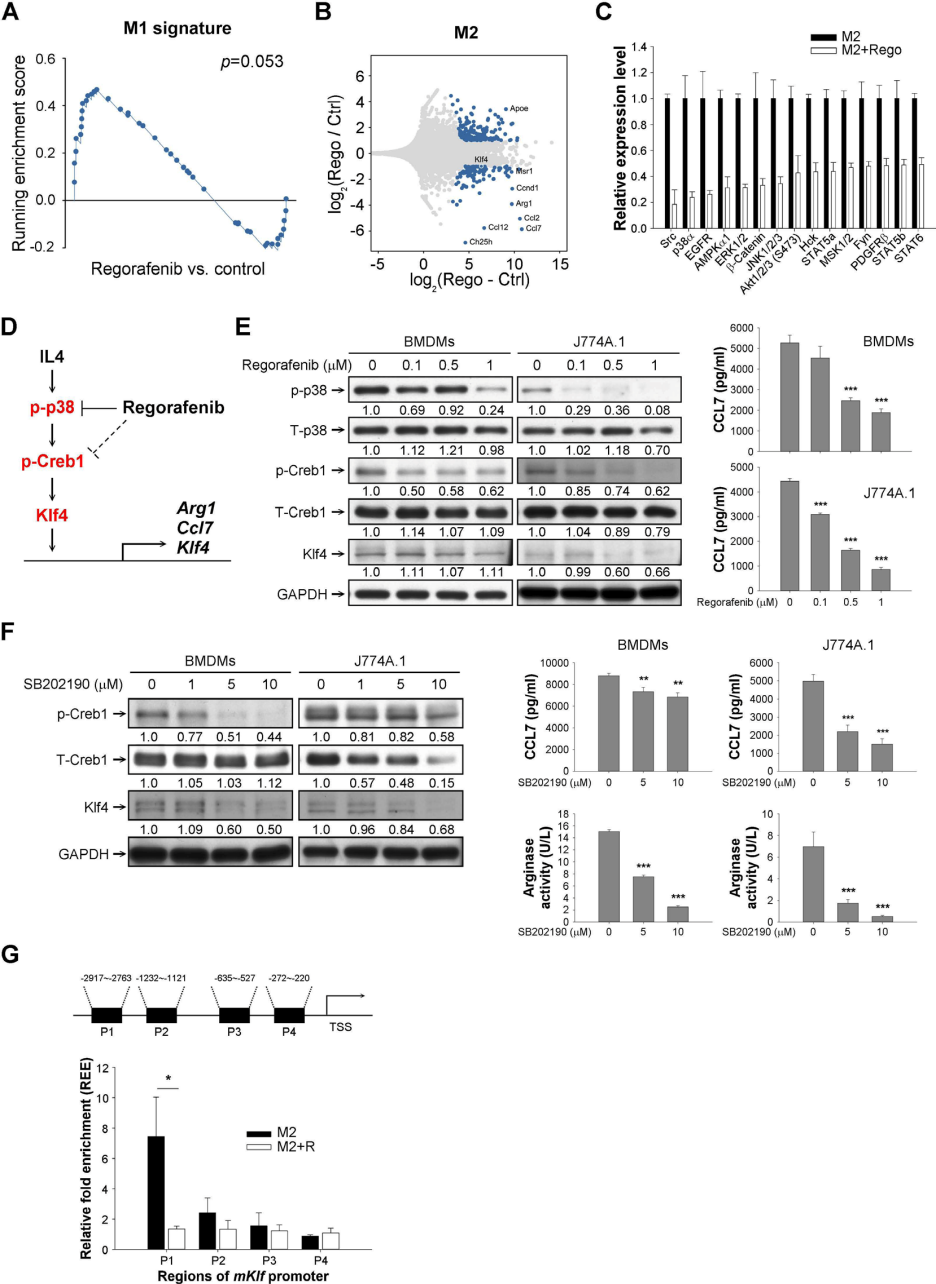
Regorafenib may regulate macrophage polarization through suppressing p38MAPK-Creb1-Klf4 pathway. (A) Gene-set enrichment analysis (GSEA) of M1 signature in regorafenib-treated or vehicle-treated M2 macrophages derived from bone marrow–derived macrophages (BMDMs). BMDMs were pretreated with regorafenib 1 µM for 1 hour, polarized to M2 phenotype, and total RNA were harvested for RNA-sequencing. (B) Representative genes regulated by regorafenib in M2 BMDMs. Genes with log (fold changes) (logFC) of >1 or <−1 were listed. (C) Inhibition of representative kinases in BMDMs by regorafenib 1 μM (phospho-kinase array). Kinases with ≥50% suppression of phosphorylation were shown. (D) A proposed mechanism by which regorafenib may prevent the M2 polarization of macrophages. (E) Suppression of p38MAPK and the downstream Creb1 phosphorylation and expression of Klf4 and CCL7 by regorafenib in macrophages. (F) Suppression of Creb1 phosphorylation, Klf4/CCL7 expression, and arginase activity in macrophages by the p38MAPK inhibitor SB202190. The number below each band in the western blot indicated the relative intensity of staining signals measured by ImageJ software (National Institutes of Health, https://imagej.nih.gov/ij/). (G) Binding of the transcription factor Creb1 to the predicted binding sites at the Klf4 promoter. Regorafenib (1 µM) significantly suppressed Creb1 binding to Klf4 promoter, particularly the −2917 to −2763 site, in M2 BMDMs. *, p < 0.05; **, p < 0.01; ***, p <0.001.

To further elucidate the molecular mechanisms by which regorafenib may suppress M2 macrophage polarization, RNA expression and activity of representative cellular kinases in BMDMs treated with regorafenib were analyzed by RNA-seq and phosphokinase array, respectively. Regorafenib increased gene expression related to M1 phenotype and suppressed multiple mediators of M2 polarization (figure 4A, B,online supplemental figure S4A, B, online supplemental table S6, S7) and may inhibit multiple cellular kinases that may involve in immune regulation (figure 4C, online supplemental figure S4C). Among the regorafenib-regulated kinases, p38MAPK was of particular interest since p38MAPK activation was recently found to promote M2 macrophage polarization^23^ in addition to its well-established roles in regulating apoptosis^24 25^ and response to oxidative stress.^26^ The colony-stimulating factor-1 (CSF-1) receptor (CSF-1R) pathway has been reported as a critical mechanism by which regorafenib exerted its immune modulatory effects,^14 27^ but this kinase was not included in the phosphokinase array we used. In both BMDMs and the J774A.1 macrophage cell line, regorafenib may inhibit CSF-1R phosphorylation at concentration of 1 µM. However, no significant changes in the expression patterns of CSF-1 response genes^28^ in BMDM were found. On the other hand, suppression of p38MAPK signaling^29^ and the downstream CREB1-responsive genes^30^ was consistent with the suppression of IL-4-responsive genes,^31^ which play critical roles in regulating M2 polarization of TAMs (online supplemental figure S4D). Therefore, p38MAPK was selected for further mechanistic exploration.

Although decreased phosphorylation of kinases such as src, EGFR, AMPK, ERK, and AKT were found by the kinase array, these kinases had much higher Kd by in vitro kinase assay with treatment of regorafenib or its active metabolites,^32 33^ suggesting that effects of regorafenib on these kinases may be secondary to the interaction in the complex signaling network in macrophages.

We next used the upstream regulator analysis tool of ingenuity pathway analysis to analyze the downregulated genes in regorafenib-treated macrophages in order to seek the potential mediator(s) regulated by regorafenib. The results revealed that multiple transcriptional factors were potentially regulated by regorafenib (online supplemental table S8), including Klf4 and cAMP response element binding protein 1 (Creb1), which have been known to be crucial in macrophage activity/survival.^34–36^ Intriguingly, p38MAPK has been noted to regulate Creb1 phosphorylation.^37^ We therefore hypothesized that regorafenib represses M2 polarization of macrophages through inhibiting p38MAPK activity, which suppresses p38MAPK-regulated Creb1 phosphorylation to downregulate *Klf4* transcription (figure 4D). Treatment of regorafenib in murine BMDMs and the J774A.1 macrophage cell line suppressed p38MAPK and Creb1 phosphorylation, as well as the expression of Klf4 and Ccl7 (a cytokine associated with M2 polarization) (figure 4E). Inhibition of the p38MAPK-Creb1-Klf4 pathway by shRNA knockdown of MAPK14 (online supplemental figure S4E, F) or the p38MAPK inhibitor SB202190 (figure 4F) showed similar effects on modulation of M2 markers. Suppression of Creb1 binding to the cAMP responsive elements of *Klf4* promoter by regorafenib validated the regulation of Creb1 binding on *Klf4* promoter by regorafenib (figure 4G).

**Figure 5:**
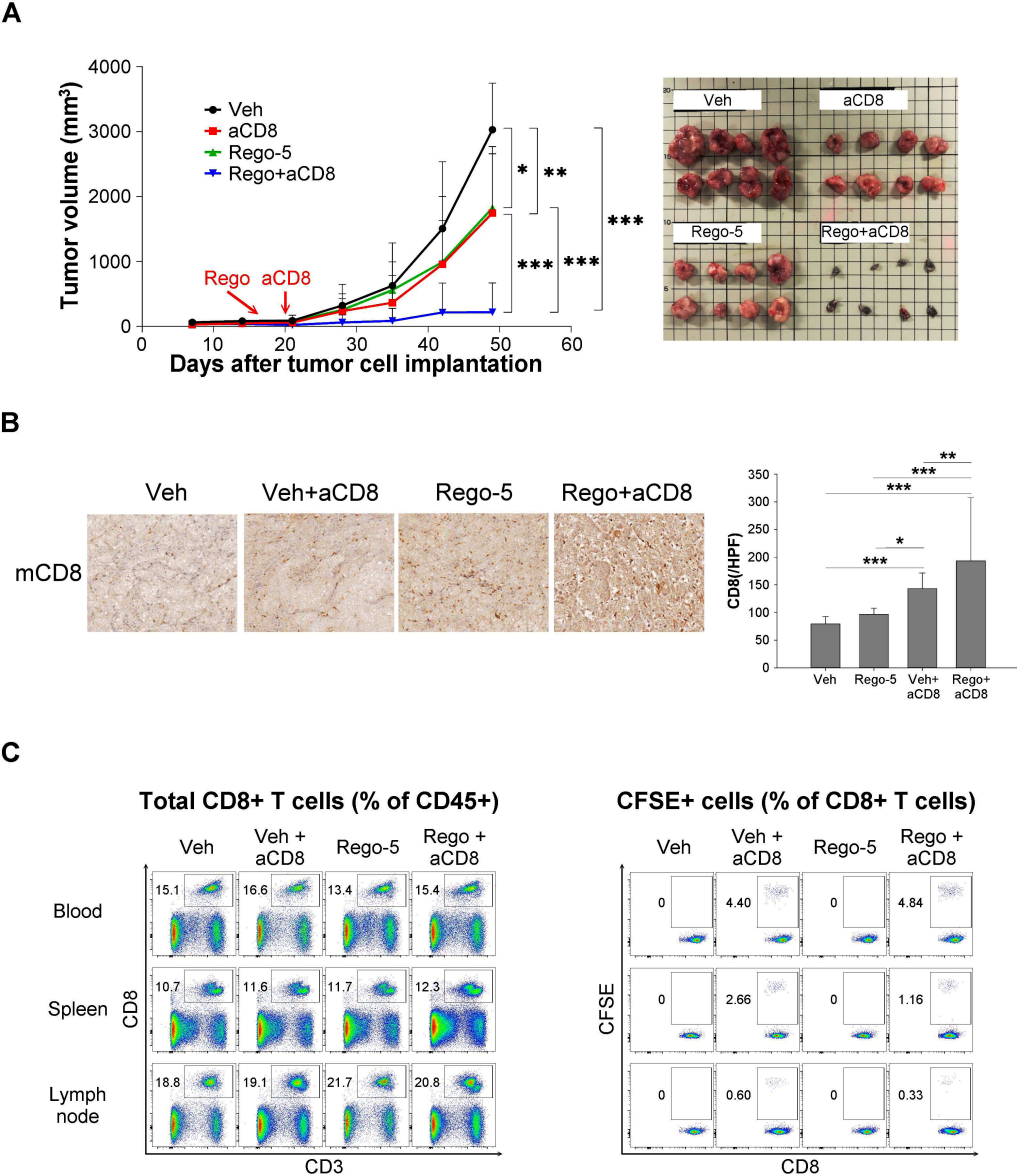
Effects of regorafenib on adoptive transfer of antigen-specific cytotoxic T cells. (A) Adoptive transfer of antigen-specific, carboxyfluorescein succinimidyl ester (CFSE)-labeled CD8+ T cells into C57BL/6 mice-bearing gp33-overexpressed Hepa1-6 cells subcutaneously. The antitumor efficacy of antigen-specific CD8+ T cells adoptively transferred into mice-bearing gp33-overexpressed tumors was enhanced by regorafenib (5 mg/kg/day) (N=8). (B) Immunohistochemistry staining and quantification of tumor-infiltrating CD8+ T cells. Data were analyzed using 20 images (regions of interest, ROI)/ tumor, 4 tumors from 4 mice in each treatment group. (C) The transferred T cells in peripheral blood, spleen, and lymph nodes were measured by flow cytometry. *, p < 0.05; **, p < 0.01; ***, p <0.001.

**Figure 6:**
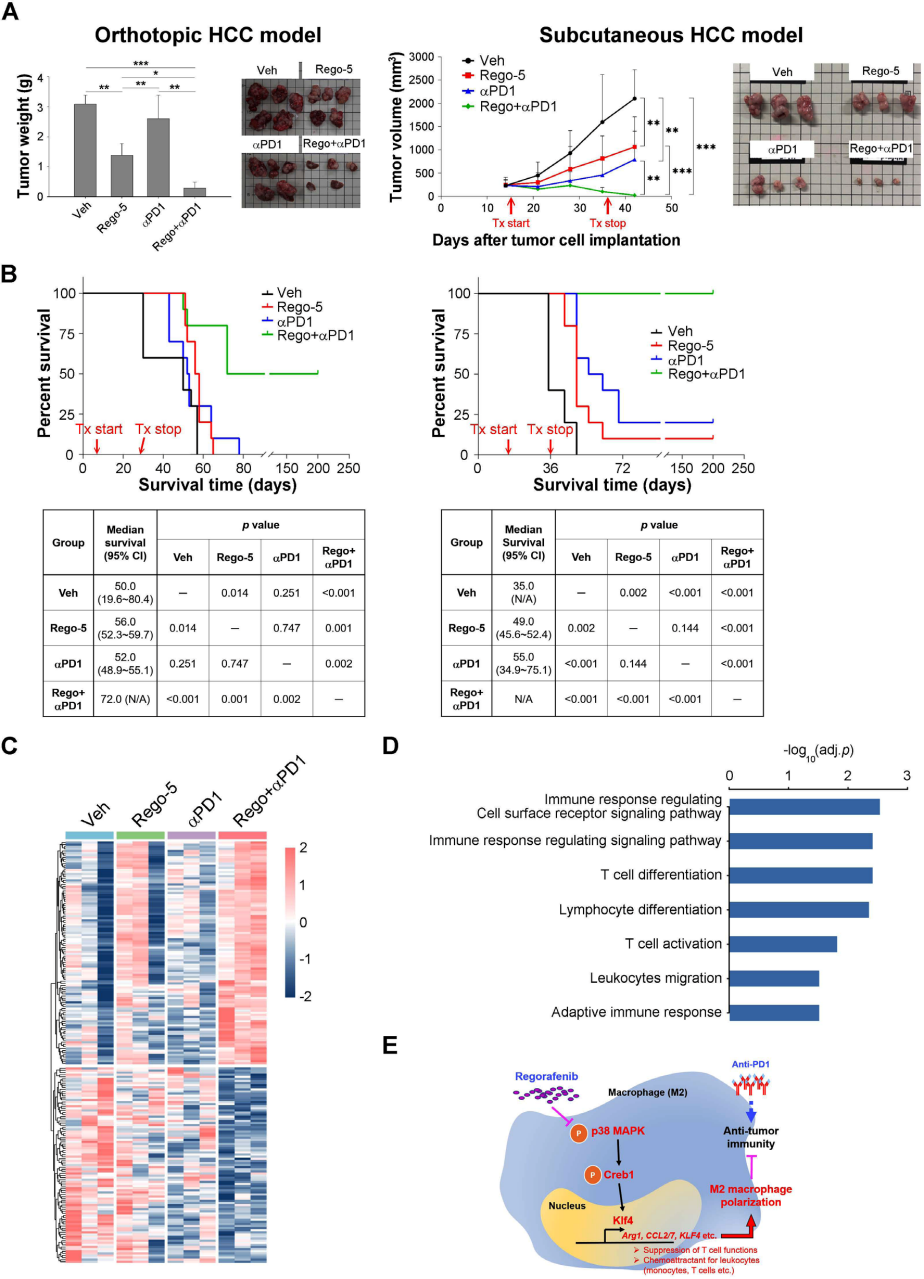
Antitumor synergy between regorafenib and anti-program cell death-1 (anti-PD1) therapy. Synergistic antitumor efficacy between regorafenib (5 mg/kg/day) and anti-PD1 (200 µg/intraperitoneal, ×5) therapy in orthotopic (BNL cell line/BALB/c mice) and subcutaneous (Hepa1-6 cell line/ C57BL/6 mice) syngeneic liver cancer models. (A) The efficacy was measured in terms of tumor weight/volume (orthotopic, N=5; subcutaneous, N=10 in each treatment group). (B) The efficacy was measured in terms of animal survival (N=10 in each treatment group). (C) Differential patterns of gene expression regulated by regorafenib and anti-PD1. Three tumors in each treatment group were subjected to RNA-seq analysis. (D) Over-representative GO terms (adj. p value<0.05) related to genes induced by the combination of regorafenib and anti-PD1. (E) Proposed mechanisms by which regorafenib regulates antitumor immunity through macrophage polarization. HCC, hepatocellular carcinoma. *, p < 0.05; **, p < 0.01; ***, p <0.001.

The effects of regorafenib on adaptive antitumor immunity were further explored by adoptive transfer of antigen-specific cytotoxic T cells and by combination with anti-PD-1 therapy. Regorafenib significantly enhanced the antitumor efficacy of the adoptively transferred CD8 T cells (figure 5A), which was associated with increased CD8 T cells in the tumors (figure 5B). On the other hand, the distribution of the adoptively transferred CD8+ T cells in peripheral blood, spleen, or lymph nodes of tumor-bearing mice did not differ with the addition of regorafenib (figure 5C). The combination of regorafenib and anti-PD1 therapy demonstrated synergistic antitumor efficacy in the liver cancer models in terms of tumor growth (figure 6A) and animal survival (figure 6B) as compared with either monotherapy. Regorafenib alone or regorafenib plus anti-PD treatment regulated multiple genes associated with leukocyte proliferation and migration in our animal models (online supplemental figure S5). Moreover, the regorafenib-anti-PD1 combination induced a distinctive pattern of gene expression, compared with treatment with either regorafenib or anti-PD1 alone (figure 6C, online supplemental table S9), and multiple immune-related pathways were involved (figure 6D, online supplemental table S10). The above data support our proposed mechanisms by which regorafenib regulates antitumor immunity (figure 6E).

## Discussion

In this study, we demonstrated that regorafenib modulates macrophage polarization and enhances antitumor immunity independent of its anti-angiogenic effects. The p38MAPK/Creb1/Klf4 signaling pathway may play a critical role in the regorafenib-induced M2 to M1 TAM polarization and subsequent T cell activation by the polarized M1 macrophages. Our study provides rationale of combining regorafenib at biologically effective dosage with other immunotherapeutic agents to improve the therapeutic index in the clinic.

While many of the immune modulatory effects of these MKIs may be linked to their VEGFR-inhibitory properties, multiple other cellular factors in the tumor microenvironment may be involved, such as increasing the M1 polarization of TAMs,^14 38–41^ enhancing CD4+ and CD8+ T cell infiltration and function,^42–45^ suppressing the number of regulatory T cells,^44 46–48^ or reversing the function of myeloid-derived suppressive cells.^40 49 50^ In this study, we demonstrated the regorafenib dosage required for immune modulation may be lower than recommended as single-agent therapy for advanced HCC treatment. The biologically effective dosage for regorafenib and other MKIs in future development of combination regimens should be clarified based on clearer understandings of molecular mechanisms.

The p38MAPK pathway is crucial for stress responses and has been implicated in a variety of pathological conditions including inflammation, aberrant apoptosis, and cancer metastasis.^51^ The Creb1 transcription factor may be responsible for many anti-inflammatory responses induced by various growth factors and inﬂammatory signals upstream of the p38MAPK pathway.^52^ Interaction of Creb1 and its downstream effector protein Klf4 in regulating macrophage function, particularly promoting M2 polarization, has also been described previously.^53 54^ The present study demonstrated that the p38MAPK/Creb1/Klf4 pathway may play a critical role in mediating the antitumor immunity induced by regorafenib. This approach may help characterize the biologically effective dosage of other MKIs and improve their therapeutic index in combination therapy. p38MAPK may also play vital roles in regulating immune modulatory effects in other cell types, such as dendritic cells,^55^ which provides additional avenues of mechanistic exploration and new target identification.

The combination of anti-PD-1/anti-PD-L1 ICI and anti-angiogenic agents has been extensively studied in multiple cancer types. In addition to advanced HCC, this strategy has also been approved for the treatment of advanced renal cell carcinoma and endometrial carcinoma.^56 57^ Multikinase inhibitors (MKIs) like regorafenib may possess VEGFR-independent effects that may contribute to antitumor immunity, at the price of higher incidence of adverse events. Although questions remain on the comparability of the immune microenvironment between preclinical models and human tumors, our study supports the feasibility of using preclinical models to identify critical immune mediators for specific MKIs and to provide mechanistic rationale of combining regorafenib at a lower dosage with anti-PD-1 ICI to improve the therapeutic index.

Many issues regarding the mechanistic regulation and functional outcome of macrophage activation/polarization require further investigation. First, in this study, macrophage polarization was identified as an angiogenesis-independent mechanism by which regorafenib exerts its antitumor immunity effects. In fact, activation of TAMs incorporates diverse and dynamic proinflammatory and anti-inflammatory signals in the tumor microenvironment, and tumor angiogenesis plays an integral part of its regulation.^58^ Therefore, regorafenib may regulate macrophage polarization in the tumor microenvironment via both angiogenesis-dependent and angiogenesis-independent mechanisms. Second, the dichotomous classification of macrophage activation (M1 vs M2) may be oversimplified. Technical advances in high-resolution analysis of macrophage phenotypes may clarify the impact of TAMs ontogeny and tissue-specific stress signals on activation and function of TAMs.^59^ Third, regorafenib may indirectly inhibit the activity of multiple kinases that regulate TAM function. For example, inhibition of adenosine 5′-monophosphate-activated protein kinase (AMPK) signaling may promote M1 polarization via metabolic reprogramming.^60^ Inhibition of AKT signaling, on the other hand, may have discrepant effects on macrophage polarization depending on the AKT isoforms involved.^61^ The CREB family of transcriptional factors may also regulate TAMs function via other targets, such as ATF4.^62^ While the complexity of TAMs activation poses formidable challenges for mechanistic exploration, it also provides enormous opportunity to find out novel immune modulatory strategy for better antitumor efficacy.

In conclusion, regorafenib may enhance antitumor immunity for HCC through modulation of macrophage polarization. This safer and biologically effective dosage of regorafenib will provide better therapeutic index for combination therapy. Optimization of preclinical models should be pursued to facilitate rational design of ICI-based combination regimens and mechanistic exploration of potential antitumor synergy among different immune modulatory agents.
