# Supplementary material for: Regorafenib enhances antitumor immunity via inhibition of p38 kinase/Creb1/Klf4 axis in tumor-associated macrophages
Source: J Immunother Cancer. 2021 Mar 22;9(3):e001657. doi: 10.1136/jitc-2020-001657 (PMC7986673; doi:10.1136/jitc-2020-001657)
Supplement: Supplementary data [file jitc-2020-001657supp002.pdf]

Supplementary materials and methods:

Cell lines

The human HCC cell lines (HepG2, Hep3B, and SNU-398) and mouse HCC cell lines (Hepa1-6 and BNL-MEA) were obtained from the American Type Culture Collection (ATCC), and the Huh-7 cell line (human HCC) was from the Health Science Research Resources Bank. The mouse macrophage cell line J774A.1 was from the Bioresource Collection and Research Center (BCRC) in Taiwan. Liver cancer cell lines and J774A.1 were cultured in Dulbecco's modified Eagle's medium (DMEM) supplemented with 10% fetal bovine serum, penicillin (100 units/mL), and streptomycin (100 μg/mL). The cells were maintained in a humidified incubator under 5% CO_2 at 37°C.

Agents used in this study

**Agents**	**Brand**
Regorafenib	Bayer
Anti-PD1	BioXcell
SB202190	Selleckchem
IFN-γ	R&D
lipopolysaccharide	Sigma

Ou D-L, *et al. J Immunother Cancer* 2021; 9:e001657. doi: 10.1136/jitc-2020-001657

IL4	R&D
carboxyfluorescein succinimidyl ester (CFSE)	Invitrogen
phospho-kinase array	R&D
TUNEL assay	Promega
Opal 4-Color Manual IHC Kit	Perkin Elmer
EasySep Mouse CD8a Positive Selection Kit II	STEMCELL

Antibodies for western blot

**Antibody**	**Brand**
p-p38	Cell Signaling
T-p38	Cell Signaling
p-CREB	Cell Signaling
T-CREB	Cell Signaling
KLF4	Santa Cruz
p-CSF-1R	Cell Signaling
T-CSF-1R	Cell Signaling
GAPDH	Santa Cruz

Antibodies for flow cytometry

**Antibody**	**Clone**	**Brand**
anti-CD45	30-F11	Biolegend
anti-IFNγ	XMG1.2	Biolegend
anti-CD11b	M1/70	Biolegend
anti-MHC II	M5/114.15.2	Biolegend
anti-CD206	C068C2	Biolegend
anti-CD335	29A1.4	Biolegend
anti-CD11c	N418	Biolegend
anti-CD123	5B11	Biolegend
anti-Gr1	RB6-8C5	Biolegend
anti-CD4	RM4-5	BD Bioscience
anti-F4/80	T45-2342 (RUO)	BD Bioscience
anti-CD3	145-2C11	ThermoFisher Scientific
anti-CD8	53-6.7	ThermoFisher Scientific
anti-CD25	PC61.5	ThermoFisher Scientific
anti-Foxp3	FJK-16s	ThermoFisher Scientific

Multiplex immunofluorescence staining

Formalin-fixed, paraffin-embedded (FFPE) tumor sections were studied by using the Polaris® system (PerkinElmer, Waltham, MA, USA) and the Opal 4-Color Manual IHC Kit (PerkinElmer), according to the manufacturer's instructions. The staining protocol used microwave treatment to remove antibodies after staining with individual antibodies and opal fluorophore (listed in supplementary method), and the process was repeated until all antigens have been stained with their respective fluorophores. Fluorescent tyramide signal amplification (TSA) reagents were used to retain intact fluorescent signals after microwave treatment. Finally, the nuclei were stained with 4′,6-diamidino-2-phenylindole (DAPI) and mounted with Prolong Diamond Antifade Mountant (ThermoFisher).

Antibodies for multiplex immunofluorescence staining.

**Antibody**	**Dilution**	**Brand**	**Opal Fluorophore**
anti-F4/80	1:100	Bioss	Opal 690
anti-MHC II	1:50	Bioss	Opal 520
anti-CD206	1:50	Bioss	Opal 570

Ou D-L, *et al. J Immunother Cancer* 2021; 9:e001657. doi: 10.1136/jitc-2020-001657

Preparation of murine bone marrow-derived macrophages (BMDMs)

Bone marrow cells were isolated from 6–8 week-old male BALB/c mice by flushing femurs and tibias of euthanized mice with DMDM/10% FBS and incubating in red blood cell lysing Buffer (BD Pharm Lyse™). The isolated bone marrow cells were cultured in DMEM supplemented 10%FBS and 20% L929-conditional medium for 7 days to differentiate to macrophages. The culture medium was replaced every three days

Chromatin immunoprecipitation (ChIP)

Bone marrow derived macrophages (BMDMs) were differentiated into M2 macrophage by IL-4, as described in the text, with or without regorafenib treatment. The cells were cross-linked with 1% formaldehyde and stopped by adding glycine to a final concentration of 0.125 M. The cells were washed by cold PBS and harvested in SDS buffer. The cells were centrifuged and the pellets were resuspended in ChIP lysis buffer for sonication. The soluble chromatin was collected by centrifugation and the supernatants were incubated with anti-CREB1 antibody rabbit mAb CST #9197 (Cell Signaling Technology, Inc. of Danvers, MA) and non-immune rabbit IgG for 1 hour at 4°C. Then Protein A magnetic beads, 30 μl, were added and the samples were incubated under gentle

agitation over night at 4°C. The DNA-protein complexes were then eluted, and the specific regions were amplified by quantitative polymerase chain reaction (qPCR).

For investigation of the direct binding of Creb1 on Klf4 promoter, we used JASPAR database to predict the Creb1 binding site on mouse *Klf4* proximal promoter region (0 to -2,945 related to transcriptional start site). The primers were designed to amplified the regions containing the probable Creb1-binding sites which contain the sequences resemble to cAMP responsive element 5'-TGACGTCA-3'.

The sequences of the primers used in ChIP experiments are:

P1 forward: 5′- CGGCGTCAGCACGGCTCCAG-3′; Reverse: 5′-CCCCCTGCCTTTCTTCTCCC-3′.

P2 forward: 5′- GCCCTCACCCCCACCCTACGG-3′; Reverse: 5′-TCCAGCCCGCCCTCTCTC-3′.

P3 forward: 5′- GCGAGTTATACTGAACACCA-3′; Reverse: 5′-AGTCTTATTACTCCTTTTCCC-3′.

P4 forward: 5′-TGGTGAGTTCAAGACCTGATT-3′; Reverse: 5′-GCACCTGCTCCTTTTCCTGGG-3′.

Ou D-L, *et al. J Immunother Cancer* 2021; 9:e001657. doi: 10.1136/jitc-2020-001657

PCR condition was set as following: 95°C, 20 sec- 95 °C, 1 sec - 60 °C, 20 sec for 50 cycles by StepOnePlus™ Real-Time PCR System (Applied Biosystems,Foster City, CA 94404. USA). The delta Ct values were calculated by the formula: Ct target gene – Ct negative control = delta Ct. The results were presented as the fold enrichments which indicate the antibodies-pulldown ChIP results relative to the IgG samples.

Schema of animal experiments in this study

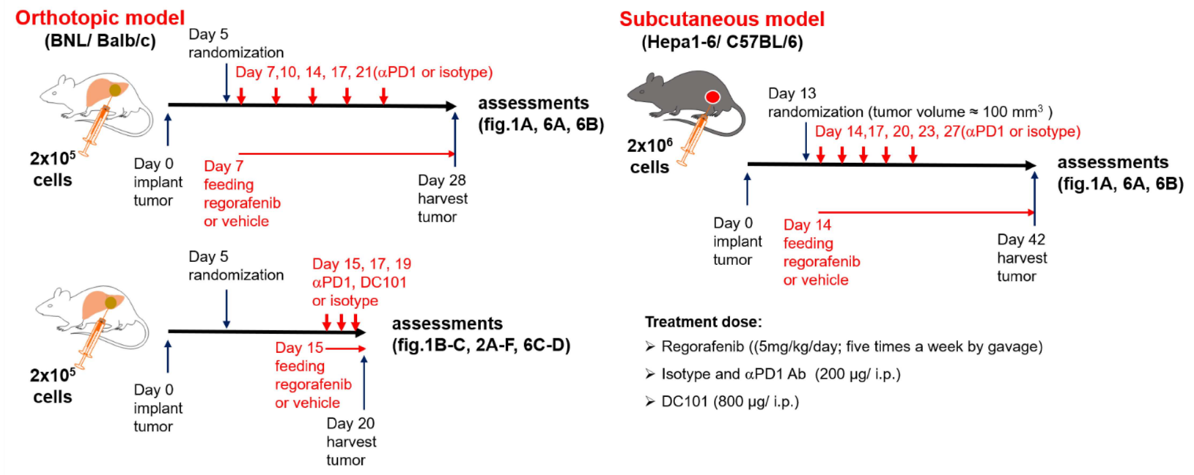

Ou D-L, *et al. J Immunother Cancer* 2021; 9:e001657. doi: 10.1136/jitc-2020-001657

Supplementary figure

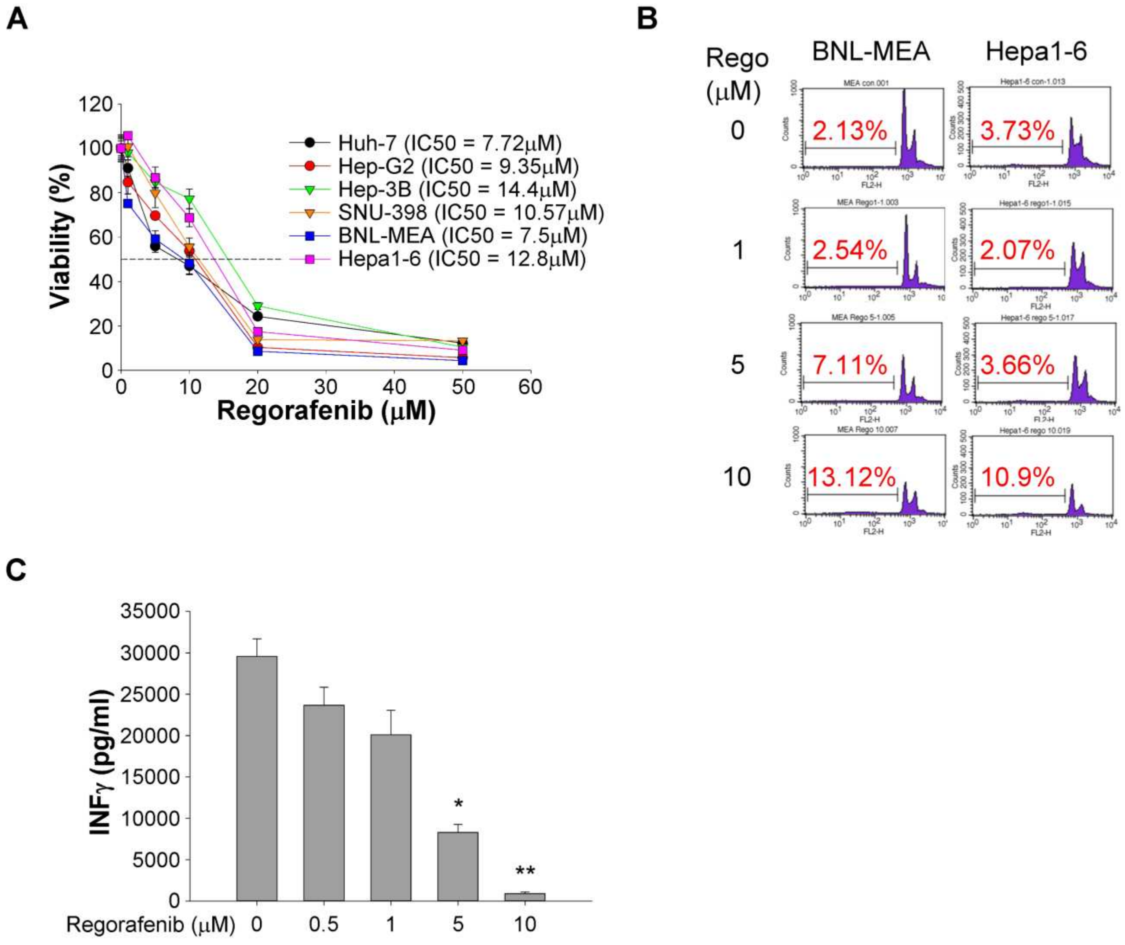

Figure S1. The immune modulatory and cytotoxic effects of regorafenib on tumor cells or immune cells.

(A) In vitro viability of liver cancer cell lines (human and murine) measured by MTT assay. (B) Regorafenib-induced apoptosis in murine liver cancer cell lines measured by flow cytometry. (C) Regorafenib may inhibit secretion of interferon-γ (IFN-γ), measured by ELISA, from murine splenocytes. *, $p < 0.05$; **, $p < 0.01$; ***, $p < 0.001$, two-tailed Student's t test.

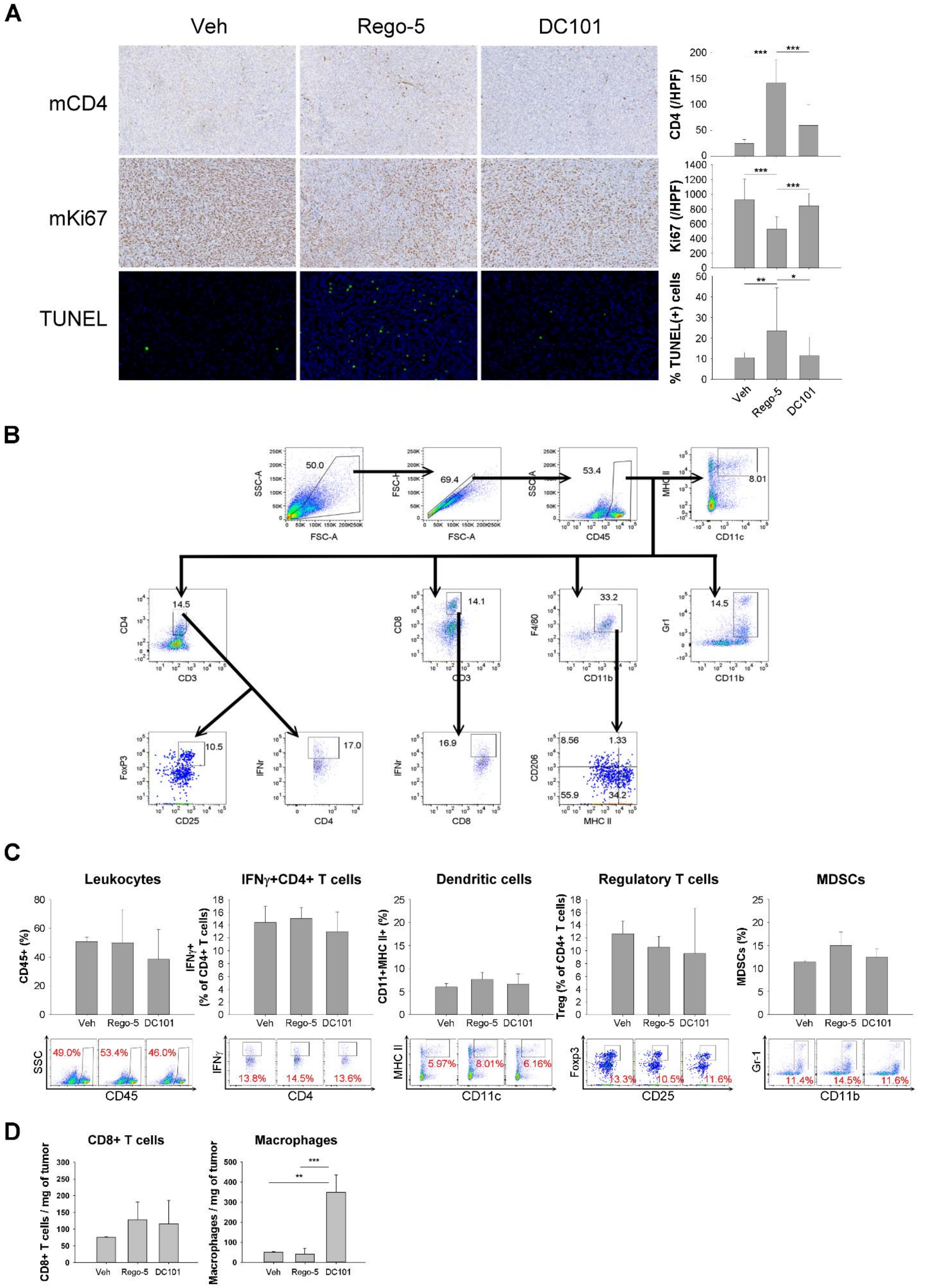

Ou D-L, *et al. J Immunother Cancer* 2021; 9:e001657. doi: 10.1136/jitc-2020-001657

Figure S2. The immune modulatory effects of regorafenib on other immune cells.

(A) Immunohistochemistry staining and quantification of tumor-infiltrating CD4+ T cells, the cell proliferation marker Ki67, and apoptosis (TUNEL assay), in tumors treated by regorafenib (5 mg/kg/day) or DC-101 (800 μg, i.p., day-1, 3, 5) for 5 days (B) Gating strategy for analysis of tumor-infiltrating immune cells by flow cytometry. (C) Composition of tumor-infiltrating immune cells after regorafenib or DC-101 treatment, analyzed by flow cytometry. (D) CD8+ T cells and macrophages of TILs after drug treatment. The bars represented mean cell number ± SD/mg tumor tissue. *, $p < 0.05$; **, $p < 0.01$; ***, $p < 0.001$, one-way ANOVA with Tukey's post hoc test.

Ou D-L, *et al. J Immunother Cancer* 2021; 9:e001657. doi: 10.1136/jitc-2020-001657

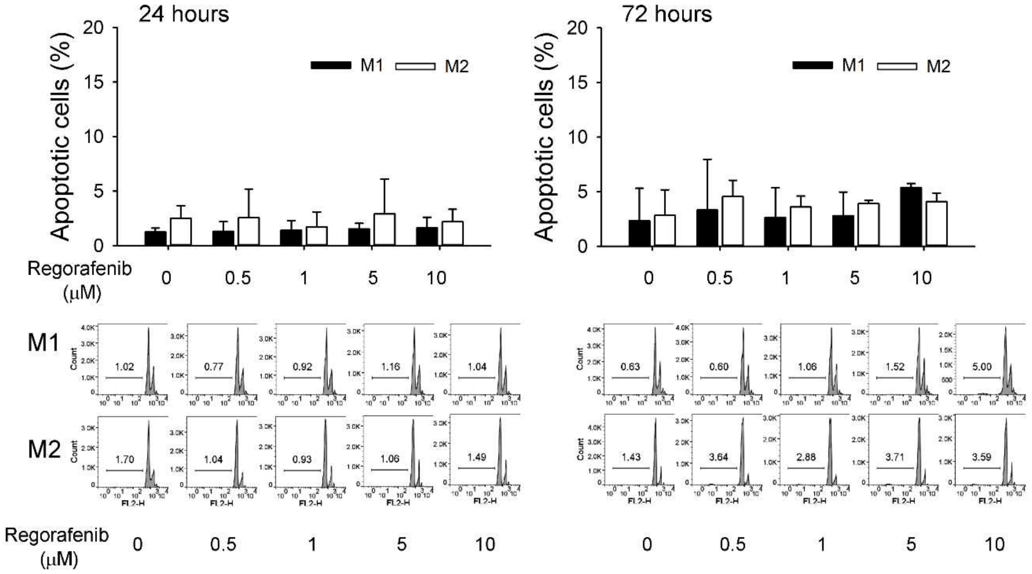

Figure S3. The cytotoxic effects of regorafenib on macrophages.

Regorafenib-induced apoptosis in murine BMDM measured by flow cytometry (sub-G1 fraction analysis). Murine BMDMs from BALB/c mice were treated with regorafenib (0, 0.5, 1, 5, 10 μM) for 24 and 72 hours, and polarized to M1 or M2 phenotypes as described methods. Columns, mean averages of three independent experiments; bars, SD.

Ou D-L, *et al. J Immunother Cancer* 2021; 9:e001657. doi: 10.1136/jitc-2020-001657

A

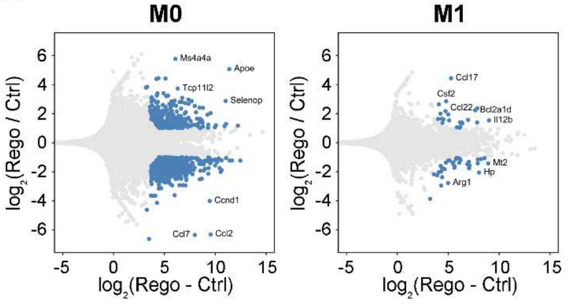

B

Genes	M2+R vs M2 logFC	M2+R vs M2 prob	Genes	M2+R vs M2 logFC	M2+R vs M2 prob
Cybb	2.29	0.93	Lipn	-1.02	0.86
Cd74	2.22	0.93	Rars	-1.08	0.86
Tbc1d16	1.15	0.86	Rgl1	-1.09	0.86
Csf2rb	1.12	0.86	Klf4	-1.09	0.86
Itgax	1.11	0.86	Bcar3	-1.10	0.86
Mxd4	1.09	0.86	Clcn5	-1.14	0.86
Sema4d	1.06	0.86	Enpp1	-1.17	0.86
Atp6v0d2	1.02	0.86	Plk2	-1.31	0.88
Tlr8	1.01	0.86	Ets2	-1.43	0.88
			Fam198b	-1.52	0.89
			Cd36	-1.88	0.92
			Mmp13	-1.91	0.91
			Serpinb9b	-2.07	0.93
			Rhob	-2.08	0.93
			Tmem26	-2.10	0.93
			Arg1	-4.01	0.97
			Ccl7	-5.95	1.00

C

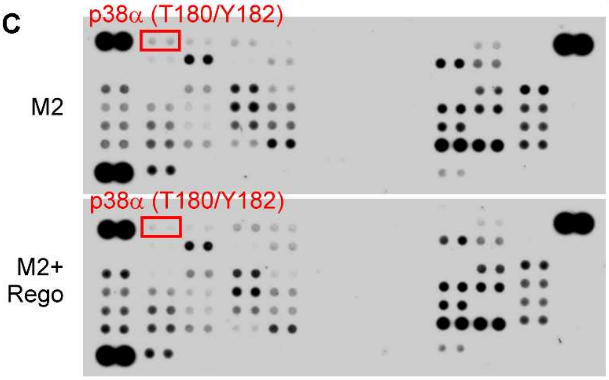

Kinase	M2+Rego vs M2
Src	18.65%
p38α	24.00%
EGFR	26.23%
AMPKα1	31.51%
ERK1/2	31.67%
β-Catenin	33.26%
JNK1/2/3	34.46%
Akt1/2/3	42.83%
Hck	43.79%
STAT5a	44.03%
MSK1/2	46.84%
Fyn	47.93%
PDGFRβ	48.50%
STAT5b	48.96%
STAT6	49.31%

Ou D-L, *et al. J Immunother Cancer* 2021; 9:e001657. doi: 10.1136/jitc-2020-001657

D

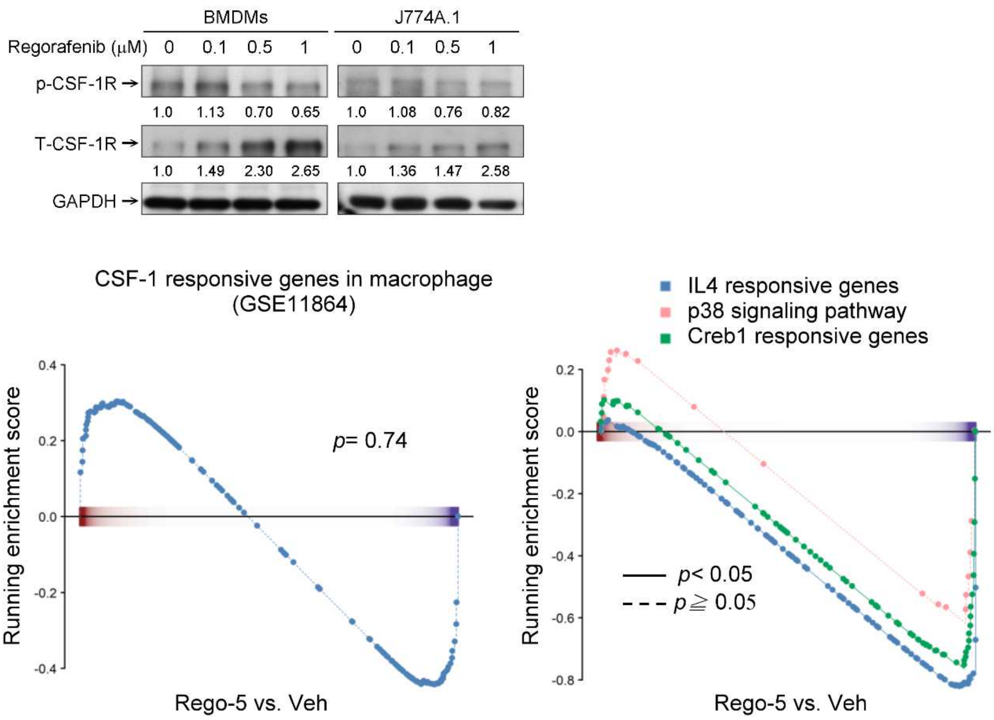

E

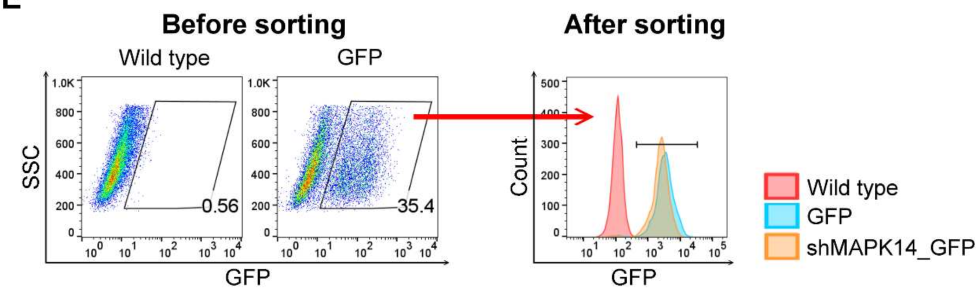

F

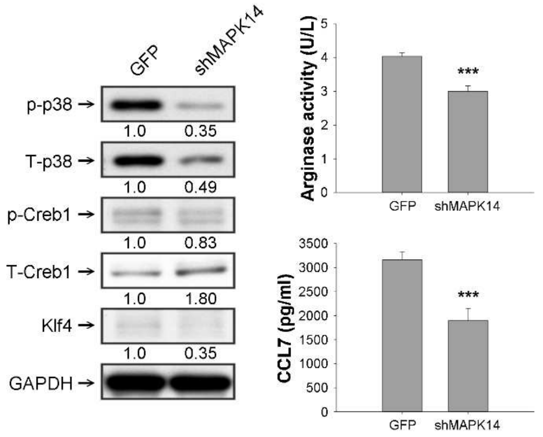

Ou D-L, et al. J Immunother Cancer 2021; 9:e001657. doi: 10.1136/jitc-2020-001657

Figure S4. Exploring the cellular mediators of regorafenib-regulated macrophage polarization.

(A) MA plots of gene expression regulated by regorafenib. BMDMs were pre-treated with regorafenib 1 μM for 1 hour, polarized to M1 or M2 phenotypes as described above, and total RNA were harvested for RNA-sequencing. (B) Fold changes of representative genes after regorafenib (1 μM) treatment in M2 BMDMs.

(C) Inhibition of representative kinases in BMDMs by regorafenib (1 μM) (phospho-kinase array). (D) In both BMDMs and the J77 macrophage cell line, regorafenib may inhibit CSF-1R phosphorylation at concentration of 1μM. However, no significant changes in the expression patterns of CSF-1 response genes (GSE11864) in BMDM.

(E) The shMAPK14-transduced J774A.1 were sorted through GFP signal and check by flow cytometry. (F) Suppression of p38MAPK /Creb1 phosphorylation, Klf4/CCL7 expression, and arginase activity in macrophages by knockdown of the upstream MAPK14 using shRNA. The number below each band in the western blot indicated the relative intensity of staining signals measured by ImageJ software (National Institute of Health, https://imagej.nih.gov/ij/).

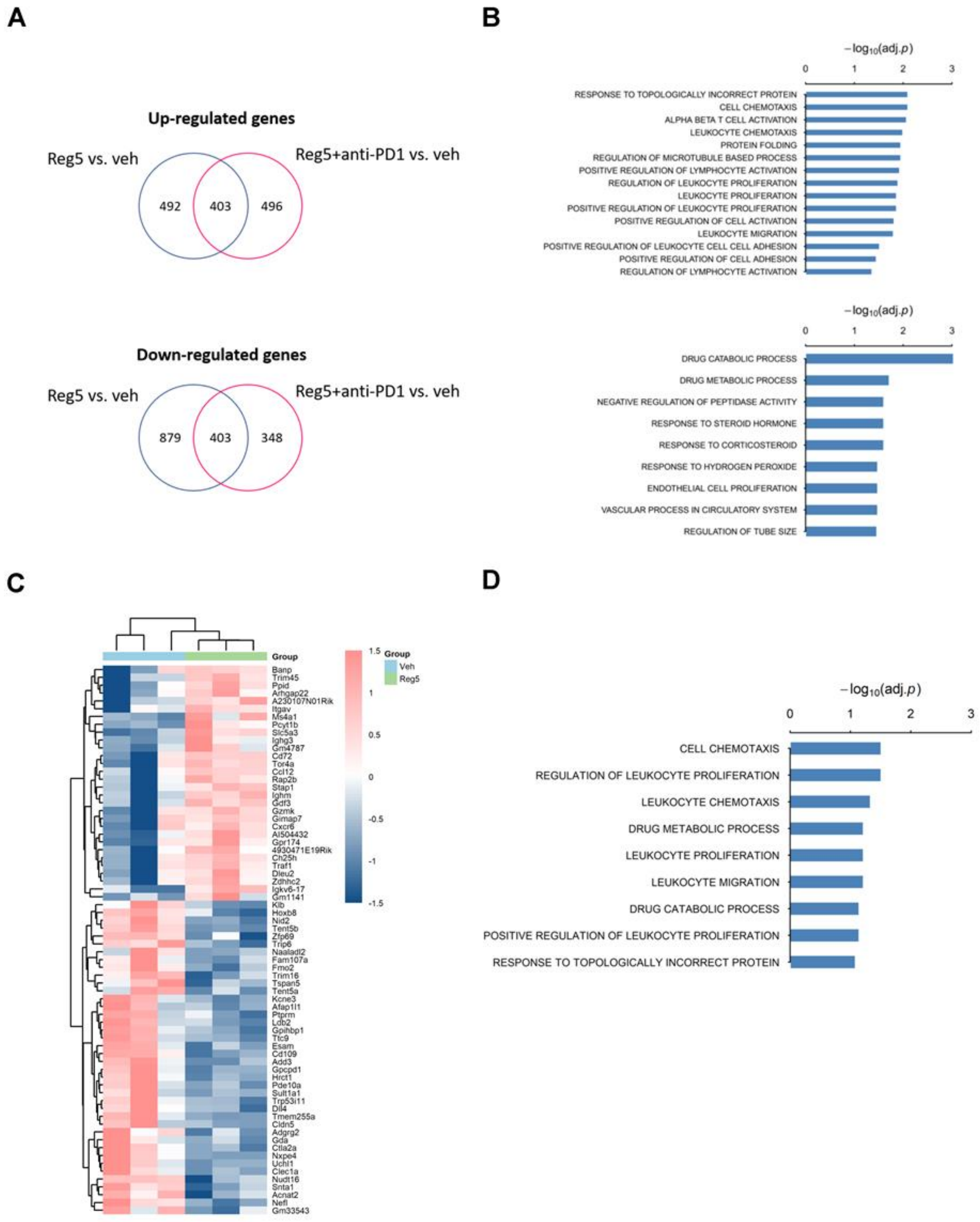
A
B
Up-regulated genes
Reg5 vs. veh
Reg5+anti-PD1 vs. veh
492
403
496
Down-regulated genes
Reg5 vs. veh
Reg5+anti-PD1 vs. veh
879
403
348
−log10(adj.p)
0
1
2
3
RESPONSE TO TOPOLOGICALLY INCORRECT PROTEIN
CELL CHEMOTAXIS
ALPHA BETA T CELL ACTIVATION
LEUKOCYTE CHEMOTAXIS
PROTEIN FOLDING
REGULATION OF MICROTUBULE BASED PROCESS
POSITIVE REGULATION OF LYMPHOCYTE ACTIVATION
REGULATION OF LEUKOCYTE PROLIFERATION
LEUKOCYTE PROLIFERATION
POSITIVE REGULATION OF LEUKOCYTE PROLIFERATION
POSITIVE REGULATION OF CELL ACTIVATION
LEUKOCYTE MIGRATION
POSITIVE REGULATION OF LEUKOCYTE CELL CELL ADHESION
POSITIVE REGULATION OF CELL ADHESION
REGULATION OF LYMPHOCYTE ACTIVATION
−log10(adj.p)
0
1
2
3
DRUG CATABOLIC PROCESS
DRUG METABOLIC PROCESS
NEGATIVE REGULATION OF PEPTIDASE ACTIVITY
RESPONSE TO STEROID HORMONE
RESPONSE TO CORTICOSTEROID
RESPONSE TO HYDROGEN PEROXIDE
ENDOTHELIAL CELL PROLIFERATION
VASCULAR PROCESS IN CIRCULATORY SYSTEM
REGULATION OF TUBE SIZE
C
D
Group
Banp
Trim45
Ppid
Arhgap22
A230107N01Rik
Itgav
Ms4a1
Pcyt1b
Slc5a3
Ighg3
Gm4787
Cd72
Tor4a
Ccl12
Rap2b
Stap1
Ighm
Gdf3
Gzmk
Gimap7
Cxcr6
AI504432
Gpr174
4930471E19Rik
Ch25h
Traf1
Dleu2
Zdhhc2
Igkv6-17
Gm1141
Klb
Hoxb8
Nid2
Tent5b
Zfp69
Trip6
Naaladl2
Fam107a
Fmo2
Trim16
Tspan5
Tent5a
Kcne3
Afap1l1
Ptprm
Ldb2
Gpihbp1
Ttc9
Esam
Cd109
Add3
Gpcpd1
Hrct1
Pde10a
Sult1a1
Trp53i11
Dll4
Tmem255a
Cldn5
Adgrg2
Gda
Ctla2a
Nxpe4
Uchl1
Clec1a
Nudt16
Snta1
Acnat2
Nefl
Gm33543
1.5
1
0.5
0
-0.5
-1
-1.5
Group
Veh
Reg5
−log10(adj.p)
0
1
2
3
CELL CHEMOTAXIS
REGULATION OF LEUKOCYTE PROLIFERATION
LEUKOCYTE CHEMOTAXIS
DRUG METABOLIC PROCESS
LEUKOCYTE PROLIFERATION
LEUKOCYTE MIGRATION
DRUG CATABOLIC PROCESS
POSITIVE REGULATION OF LEUKOCYTE PROLIFERATION
RESPONSE TO TOPOLOGICALLY INCORRECT PROTEIN

Figure S5. Exploring the common immune modulatory mechanisms between regorafenib alone and regorafenib-anti-PD1 combination in animal models.

(A) Overlapping genes which significantly up- (top) or down-regulated (bottom) under the treatment of regorafenib alone and combine with anti-PD1. RNA seq analysis between regorafenib (5 mg/kg/day) and combine with anti-PD1 (200 μg/ i.p. x5) therapy in orthotopic (BNL cell line/BALB/c mice) syngeneic liver cancer models. (B) Bar plots depict the over-representative GO terms (adj. p value < 0.05) of the commonly up-(top) or down-regulated (bottom) genes with the treatment of regorafenib alone and combine with anti-PD1. (C) Heatmap shows the genes which are illustrated in (A) and also differentially expressed under the treatment of regorafenib in subcutaneous syngeneic liver cancer model. RNA seq analysis with regorafenib (5 mg/kg/day) therapy in subcutaneous (Hepa1-6 cell line/ C57BL/6 mice) syngeneic liver cancer model. (D) Over-representative GO terms (adj. p value < 0.1) of genes illustrated in (C).

Ou D-L, *et al. J Immunother Cancer* 2021; 9:e001657. doi: 10.1136/jitc-2020-001657

Supplementary tables

Supplementary table S1 Curated gene sets used in this study

Supplementary table S2 GSEA of regorafenib (related to Figure 1C)

Supplementary table S3 Transcriptomic analysis of regorafenib- and DC101-treated tumor in orthotopic HCC model (related to Figure 2B)

Supplementary table S4 GO term analysis of regorafenib-specific responsive genes in orthotopic HCC tumor (related to Figure 2C)

Supplementary table S5 GSEA analysis of immune-related pathway in tumor from orthotopic HCC model (related to Figure 2F)

Supplementary table S6 GSEA analysis of regorafenib-treated in M2 BMDMs (related to Figure 4A)

Supplementary table S7 Transcriptomic analysis of regorafenib-responsive genes in BMDMs (related to Figure 4B)

Supplementary table S8 prediction of TF and its binding site by IPA and online database

Supplementary table S9 Transcriptomic analysis of vehicle-, regorafenib, anti-PD1, or regorafenib + anti-PD1 treated tumor in orthotopic HCC model

Supplementary table S10 GO term analysis related to combination-responding genes

Ou D-L, *et al. J Immunother Cancer* 2021; 9:e001657. doi: 10.1136/jitc-2020-001657
